# The Role of Microglia in Alzheimer’s Disease From the Perspective of Immune Inflammation and Iron Metabolism

**DOI:** 10.3389/fnagi.2022.888989

**Published:** 2022-06-30

**Authors:** Hui-Zhi Long, Zi-Wei Zhou, Yan Cheng, Hong-Yu Luo, Feng-Jiao Li, Shuo-Guo Xu, Li-Chen Gao

**Affiliations:** ^1^School of Pharmacy, The Affiliated Changsha Central Hospital, Hengyang Medical School, University of South China, Changsha, China; ^2^Hunan Provincial Key Laboratory of Tumor Microenvironment Responsive Drug Research, Hengyang, China

**Keywords:** Alzheimer’s disease, microglia, iron metabolism, Aβ, immune inflammation

## Abstract

Alzheimer’s disease (AD), the most common type of senile dementia, includes the complex pathogenesis of abnormal deposition of amyloid beta-protein (Aβ), phosphorylated tau (p-tau) and neuroimmune inflammatory. The neurodegenerative process of AD triggers microglial activation, and the overactivation of microglia produces a large number of neuroimmune inflammatory factors. Microglia dysfunction can lead to disturbances in iron metabolism and enhance iron-induced neuronal degeneration in AD, while elevated iron levels in brain areas affect microglia phenotype and function. In this manuscript, we firstly discuss the role of microglia in AD and then introduce the role of microglia in the immune-inflammatory pathology of AD. Their role in AD iron homeostasis is emphasized. Recent studies on microglia and ferroptosis in AD are also reviewed. It will help readers better understand the role of microglia in iron metabolism in AD, and provides a basis for better regulation of iron metabolism disorders in AD and the discovery of new potential therapeutic targets for AD.

## Introduction

Alzheimer’s disease (AD) is a neurodegenerative disease that is the most common form of dementia, presenting clinically as memory loss and cognitive decline. This disease is characterized by extracellular deposition of amyloid beta-protein (Aβ) in senile plaques and neurofibrillary tangles (NFTs) within neurons composed of hyperphosphorylated tau proteins. These events are accompanied by neuronal and synaptic loss, chronic inflammation, and oxidative stress (Lane et al., 2018; Caruso et al., 2021). The exact mechanisms that lead to these changes in AD are still inconclusive, and whether they are causative factors or consequences of the disease is debated. Among many hypotheses, immune inflammation has been suggested as an important mechanism for progressive neuronal degeneration and death in AD, in addition to the widely accepted Aβ hypothesis and the tau protein hypothesis (Walker et al., 2015; Yeh et al., 2016; Jackson et al., 2020). And each hypothesis is not independent, and a growing body of evidence supports a direct relationship between abnormal Aβ production and neuroimmune inflammation and oxidative stress (Guerriero et al., 2017). Typically, AD is divided into early-onset and late-onset AD (also known as sporadic AD), the latter accounting for approximately 90% of individuals with AD (Mendez, 2017). The development of early-onset AD is related to altered genetic background (Lanoiselée et al., 2017). Although numerous risk factors are associated with the development of late-onset AD, the complete mechanism of its development remains unclear. These factors include: genetic factors such as the presence of the apolipoprotein E4 (APOE4) allele, environmental factors and some lifestyle factors (Vos et al., 2017).

Iron homeostasis is closely related to the pathology of Alzheimer’s disease. Emerging evidence suggests that iron is involved in the deposition of Aβ plaques and the formation of NTFs (Hare et al., 2013). In AD, iron is observed to be present in localized areas of neuronal death, which further supports the metal theory of dementia that iron promotes neurodegeneration (Bush, 2013). Iron, as a redox-active transition metal, plays a key role in inducing oxidative stress, which ultimately leads to neuronal cell death. This oxidative stress is thought to be exacerbated by the induction of neuroinflammation (Thomsen et al., 2015). Iron homeostasis genes [Hemochromatosis (HFE: C282Y, H63D)] are associated with a high risk of AD (Tisato et al., 2018). On the other hand, studies have shown that the C282Y and H63D HFE variants increase the risk and severity of AD under the synergistic effect of transferrin gene polymorphisms (Lehmann et al., 2012). Classic iron-responsive mRNA element (IRE) genes are sufficiently sensitive to iron overload and iron deficiency. Studies have found that iron regulatory proteins (IRPs)/IRE system interfered by iron accumulation promotes amyloid precursor protein (APP) level increase, Aβ deposition and neuron loss of AD (Yao et al., 2022). A study revealed changes in gene expression associated with the IRP/IRE system in the brain of young PSEN1 fAD mutation carriers, which is consistent with the idea that iron homeostasis disorder is an early contributor to the disease process (Hin et al., 2021). Interestingly, iron overload also leads to a new type of iron-dependent cell death-ferroptosis. Efforts have been made to understand the changes in neurons in AD in the presence of iron disorders and ferroptosis.

In the central nervous system (CNS), microglia are commonly involved in supportive roles, including promoting energy metabolism, synaptic plasticity, and ion homeostasis. Microglia are also the resident immune cells in the brain. Microglia activation is usually triggered by neurodegenerative processes, and it is of great interest to study their role in neurological diseases. Genome-wide association studies (GWAS) have identified 30 genetic risk loci for AD, many of which are associated with microglial function, such as APOE and triggering receptor expressed on myeloid cells 2 (TREM2) variants (Efthymiou and Goate, 2017; Kunkle et al., 2019; Nguyen et al., 2020). Inflammation in the brain is mediated by microglia and astrocytes, which generate inflammatory stimuli in response to signals (Liddelow et al., 2017). In AD, a persistent inflammatory state may lead to nerve damage followed by neuronal cell death, thereby promoting the secretion of pathological forms of tau into the extraneural milieu. In contrast, tau oligomers can reactivate microglia and cause persistent damage to neuronal cells (Morales et al., 2014; Maccioni et al., 2018). Furthermore, microglia express a variety of iron-related proteins, which are closely related to the uptake and metabolism of iron (Bishop et al., 2011; van Duijn et al., 2017; Song et al., 2018). And microglia are part of the blood-brain barrier (BBB), which limits iron uptake in healthy brains. However, in the disease state, changes in BBB permeability lead to alterations in brain iron metabolism (Rosenblum and Kosman, 2022). Excessive accumulation of iron ions will in turn affect brain iron metabolism (Brian et al., 2018). Clearly, microglia and iron metabolism are important for disease remission or progression.

Therefore, we focused on the effect of microglia on iron homeostasis in the neurodegenerative disease-AD, and reviewed the interactions between microglia, iron and immune inflammation. Finally, we also reviewed the recent progress of microglia and ferroptosis research in AD. Therefore, it is convenient for readers to better understand the role of microglia in iron metabolism in AD.

## Microglia: An Important Role in Neuroimmune Inflammation

The human body can defend itself against pathogenic invasion as well as endogenous damage through adaptive and innate immune systems (McManus, 2022). The initiation of the immune system is largely dependent on various macrophages (including microglia), which express pattern recognition receptors (PRRs). Activation of these PRRs triggers a rapid signal transduction pathway that releases cytokines and chemokines, affecting cellular function (McManus, 2022). Neuroinflammation can be defined as the response of the CNS to exogenous and/or endogenous factors that interfere with normal cellular homeostasis (Maccioni et al., 2018). In the CNS, microglia recognize a wide range of endogenous and exogenous stimuli. Among these stimuli, inflammatory signals can be classified as danger-associated molecular patterns (DAMPs), pathogen-associated molecular patterns (PAMPs), or homeostasis-altering molecular processes (Schafer et al., 2012). In addition to astrocytes, microglia are also involved in the entire process from innate immunity to subsequent inflammation in the brain (Maccioni et al., 2018; Pang et al., 2022; Van Zeller et al., 2022). Therefore, we summarize this process here with the concept of neuroimmune inflammation.

Microglia originate from primitive macrophages in the yolk sac, and are one of the key regulators of the innate immune response in the brain (Gomez Perdiguero et al., 2015). The functions of microglia are multifaceted, including synaptic pruning and remodeling during development, monitoring to clear metabolites and degenerated tissue components in the normal CNS, as well as degrading substances such as pathogenic factor Aβ plaques or harmful viruses and bacteria in diseased states (Heneka et al., 2015b; Cangalaya et al., 2020; Favuzzi et al., 2021). Microglia, for example, regulate neuronal plasticity by monitoring and pruning synapses in the adult brain (Weinhard et al., 2018). Synaptic regulation by microglia can facilitate adaptation to the environment through the formation or remodeling of memory synapses (Wang et al., 2020). In addition, microglia play an important role in regulating myelination, controlling and maintaining vascular integrity, neurogenesis, and astrogenesis (Lampron et al., 2015; Diaz-Aparicio et al., 2020; Dudiki et al., 2020). Microglia act as primary phagocytes in the brain, scavenging dead cells, cellular debris and other harmful inflammatory stimuli from the healthy brain (Damisah et al., 2020; Diaz-Aparicio et al., 2020). Microglia detect DAMPs and PAMPs through PRRs, including Toll-like receptors (TLRs), nucleotide binding oligomeric domain (NOD)-like receptors (NLRs), and absent in melanoma 2 (AIM2)-like receptors (Freeman et al., 2017; Ma et al., 2021). Upon detection of DAMPs or PAMPs, activated microglia produce and release a series of cytokines and chemokines to elicit an immune response and respond to the microenvironment following injury or pathological events (Wang et al., 2021).

There are three common microglial phenotypes (Clayton et al., 2017). Under healthy and non-aged conditions, microglia in the adult brain exhibit a homeostatic phenotype. During normal aging, the expression of homeostasis markers gradually decreases, resulting in reduced functions including proliferation, phagocytosis, branching, and cytokine secretion, at which point microglias exhibit a dystrophic phenotype (von Bernhardi et al., 2015; Clayton et al., 2017). Even worse than the dystrophic phenotype are the disease-associated microglia (DAM) associated with neurodegeneration (Keren-Shaul et al., 2017). In a gene expression profile study, DAM showed decreased expression of homeostasis microglia genes and upregulation of genes that regulate inflammation (Krasemann et al., 2017). With the development of AD, microglia can be transformed from homeostasis microglia into DAM located around Aβ plaques (Keren-Shaul et al., 2017). In the healthy brain, resting microglia cells exhibit dynamic behavior, monitoring their environment (Clayton et al., 2017). When changes in brain homeostasis are detected, microglia rapidly change shape and function in response to changes in their microenvironment, which is called microglial activation (Choi et al., 2012; Cherry et al., 2014). The classification of microglial activation is often referred to the classical macrophage classification, which classifies the microglia activation state as M1 or M2 (Colonna and Butovsky, 2017; Hanslik et al., 2021). Although the scope of the M1/M2 microglia classification has recently been controversial, the classification may help to explain the pathological relationship between inflammation and degenerative CNS diseases (Song and Suk, 2017; Guo et al., 2022). Therefore, we still use the polarization of microglia from M1 to M2 phenotype to discuss the role of microglia in AD.

M1 activation is pro-inflammatory and neurotoxic, mainly caused by activation of TLR and interferon γ (IFN-γ) signaling pathways. In this state, microglia synthesize and secrete pro-inflammatory cytokines and chemokines, such as tumor necrosis factor-α (TNF-α), interleukin-6 (IL-6), IL-1β, IL-12, and some members of chemokine (C-C motif) ligand 2 (CCL2). Microglia also express inducible nitric oxide synthase (iNOS), which converts arginine to NO (Colonna and Butovsky, 2017). The accumulation of NO increases the neurotoxic effects of glutamate (Colonna and Butovsky, 2017). In contrast, microglia in the M2 state release anti-inflammatory cytokines such as IL-4, IL-10, IL-13, transforming growth factor-β (TGF-β). Besides, M2 microglia also induce arginase 1 to facilitate the conversion of arginine to polyamines. These cells secrete insulin like growth factor I (IGF-I), fibroblast growth factor (FGF), and neurotrophic factors that promote repair and phagocytosis (Zhu et al., 2019). Upon recognition of stimulatory signals, microglia are activated. Moderately activated microglia are in a dynamic balance between M1 and M2 phenotypes, which can respond quickly to injurious stimuli. However, when brain tissue is over-stimulated, the over-activated microglia are converted into M1 phenotype, which regulates inflammatory factors and damage neurons. The damaged neurons release a variety of toxic contents, such as Aβ, which further induce microglia to form an M1 phenotype and release neuroimmune inflammatory factors again (Xie et al., 2020). Thus, a vicious cycle develops between damaged neurons and M1 phenotypic microglia. Obviously, excessive neuroimmune inflammation damages neurons, ultimately leading to neuronal death and brain tissue atrophy (Saito and Saido, 2018).

## Neuroimmune Inflammation: Pathology of Alzheimer’s Disease

Extracellular Aβ deposits and accumulation of intracellular NFTs are hallmarks of Alzheimer’s disease. Aβ is generated by hydrolysis of APP, and subsequently forms Aβ oligomers that aggregate into senile plaques. Tau is a microtubule-stabilizing protein, but aggregated hyperphosphorylated tau (p-tau) forms NFTs (Lane et al., 2018; Caruso et al., 2021). In the study of the pathogenesis of AD, scholars have suggested that inappropriate activation of immunity and inflammation may be important in the pathogenesis of AD. Elevated expression of markers associated with innate and adaptive immune system responses has been found in AD mouse models as well as in the brains of AD patients (Zotova et al., 2013). GWAS have identified risk loci for AD pathogenesis that are closely associated with microglia genes (McQuade and Blurton-Jones, 2019). Moreover, epidemiological investigations found that the prevalence of AD was significantly lower in people taking long-term non-steroidal anti-inflammatory drugs than in the general population, suggesting that the development of AD is associated with neuroinflammation (McGeer and McGeer, 2013; Wang et al., 2015). Chronic neuroinflammation and glial cell activation accompany AD pathology and partially mediate Aβ plaques and NFTs. Studies have shown that pro-inflammatory gene polymorphisms such as CCL3/macrophage inflammatory protein 1 (MIP-1α) and IL-6 have been identified as risk factors for AD. While activated microglia also produce CCL3/MIP-1α and IL-6 (Zhao et al., 2018). Translocator protein (TSPO) is a mitochondrial outer membrane protein. Several studies have used TSPO tracers (including 11C-PK11195 and 18F-flortaucipir) to show dual-phase inflammatory trends. The results showed that early microglia activation was associated with an increase in Aβ load as well as tau protein accumulation. When the Aβ load reached a plateau, microglia activity subsequently decreased (Fan et al., 2017; Ismail et al., 2020). It was found that cerebrospinal fluid soluble TREM2 levels in AD patients increased significantly with the progression of the disease. Notably, TREM2-dependent microglia activity slows down amyloid plaque formation in the early stages of AD, but not in the late stages of AD (Parhizkar et al., 2019). In the brains of AD patients, a large number of activated microglia are clustered around the neuroinflammatory plaques, exerting their phagocytic function and constituting the first line of defense against the immune responses (Saito and Saido, 2018). This suggests that microglia are predominantly activated as M2 phenotype in early AD, which facilitates the inhibition of Aβ deposition and the aggregation of NFTs (Kulkarni et al., 2022). Thus, neuroimmune inflammation is beneficial in the early stages of AD. However, with the increasing number of neuritic plaques and NFTs in the brains of AD patients, deposited Aβ and p-tau can activate microglia receptors, generate neuroimmune inflammatory responses, and accelerate the degeneration and death of neurons, ultimately leading to cognitive impairment and dementia (Maccioni et al., 2018).

Bacterial endotoxin lipopolysaccharide (LPS), an inflammatory source produced by gram-negative bacteria, has a strong immunogenicity and highly pro-inflammatory effect on human neurons. It has been reported that large amounts of LPS have been detected in the neocortex and hippocampus of AD brains (Zhao et al., 2017a,b). LPS promotes an increase in γ-secretase, leading to a large accumulation of Aβ. On the other hand, LPS-induced neuroimmune inflammation can lead to tau hyperphosphorylation and the formation of NFTs after continuous aggregation (Anthony et al., 2006). In addition, LPS further induces classical microglia-mediated innate immune and inflammatory responses by activating transmembrane protein receptors expressed in microglia (Zhao et al., 2017a).

The pathogenesis of AD is closely related to oxidative stress (Bonda et al., 2010; Di Bona et al., 2010). Evidence suggests that levels of damaging ROS are significantly higher in the AD brain than in healthy brains (Di Bona et al., 2010; Lane et al., 2018). In AD, oxidative stress influences microglia morphology changes and enhances microglial activation by promoting neurotoxic oligomerization of Aβ peptide and Tau tangles (Peters et al., 2018). Importantly, long-term stimulation of microglia by Aβ induces chronic inflammation (Heneka et al., 2015a). This may be partly due to the fact that microglia express a variety of receptors (including CD36, TLR2, TLR4, etc.) that bind to Aβ and induce proinflammatory effects (Su et al., 2016). In addition, Aβ triggers inflammasome-related signaling in microglia, activates IL-1β and IL-18 via NLR family pyrin domain containing 3 (NLRP3)/apoptosis-associated speck-like protein containing a CARD (ASC)/Caspase1, thereby promoting AD progression through deleterious inflammatory responses (Yang et al., 2019; Zhao et al., 2020). β-Nicotinamide adenine dinucleotide phosphate oxidase (NOX) is the only known enzyme family that specifically produces reactive oxygen species (ROS). NOX2 is the initiator of neuroinflammation-mediated progressive neurons degeneration. During inflammation, NOX2, which is responsible for superoxide production, is overactivated in immune cells, including microglia (Siegel et al., 2019). When NOX2 is stimulated to produce superoxide, which damages neurons, it can also activate NF-κB, which in turn produces a large number of inflammatory factors through various signal transduction pathways, and may lead to neuronal death in pathological conditions (Haslund-Vinding et al., 2017; Sun et al., 2021).

## The Role of Microglial in Alzheimer’s Disease

**FIGURE 1:**
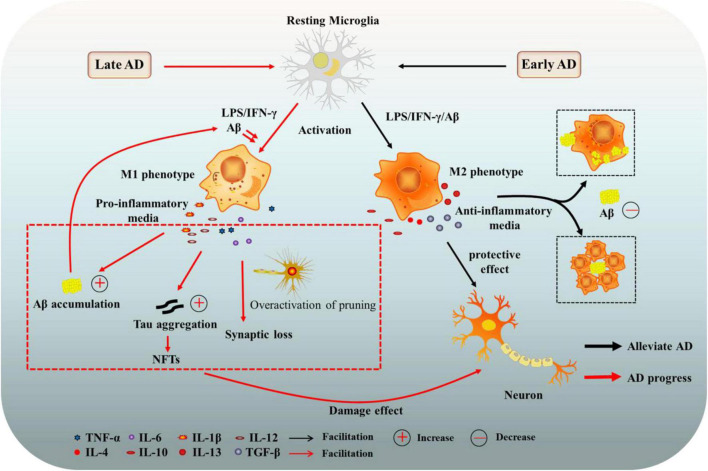
The role of microglia in Alzheimer’s disease (AD). Microglia can have beneficial or harmful effects on AD. In the early stage of AD, the activation state of microglia is mainly a protective phenotype (M2), secreting anti-inflammatory mediators and neurotrophic factors, and clearing or isolating Aβ and Tau proteins, contributing to the protection of neurons. In late AD, microglia tend to be activated as a harmful phenotype (M1), enhancing the release of pro-inflammatory factors, which damage neurons and promote the progression of AD. AD, Alzheimer’s disease; Aβ, amyloid beta-protein; NFTs, neurofibrillary tangles; LPS, lipopolysaccharide; TGF-β, transforming growth factor-β; IFN-γ, interferon γ; TNF-α, tumor necrosis factor-α; IL-6, interleukin-6.

Microglia have a wide range of physiological functions and are closely related to the occurrence and development of neurodegenerative diseases (Kulkarni et al., 2022). Changes in microglia were once thought to be a secondary event in the development of AD. However, it is now clear that microglia, while not the primary initiator of AD, play an important cellular autonomous role in the disease. Immunohistochemical analysis revealed that microglia surround amyloid plaques in both human and animal AD models, which might be beneficial or detrimental in preventing disease progression (Hansen et al., 2018). Based on a longitudinal study of human brain images, Fan et al. (2017) pointed out that the activation state of microglia shifts from an early protective phenotype to a late harmful phenotype during the development of AD. And we briefly illustrated this relationship in Figure 1.

### Microglia-Associated Immune Receptors and Alzheimer’s Disease

The immune-related receptors expressed in microglia are involved in immune surveillance, recognition and presentation of antigens, phagocytosis of harmful substances such as Aβ, and secrete immune effectors, which are important for maintaining neurological homeostasis during AD. Microglia are known to express a variety of TLRs (Su et al., 2016; Duan et al., 2021). Toll-like receptors TLR2 and TLR4 induce M1 microglia activation and neurodegeneration during AD (Su et al., 2016). Study reported that Aβ activates microglia via TLR2 and produces neuroimmune inflammatory factors including TNF-α, IL-6, and IL-1β (Qin et al., 2018). While TLR9 exerts anti-inflammatory and anti-apoptotic effects through the activation of p38 mitogen-activated protein kinase (p38MAPK) and extracellular regulatory protein kinase (ERK) pathways (Su et al., 2016; Duan et al., 2021). Advanced glycosylation end-product-specific receptor (AGER) is a member of the immunoglobulin superfamily that is expressed on the cell surface, and can bind to ligands such as AGE, Aβ and synaptic growth factor to activate microglia, induce the production of inflammatory factors, NO, ROS and other substances, and trigger a cascade reaction to aggravate AD (Maclean et al., 2019).

Genetic variants of microglia immune-associated receptors have been shown to have significant genetic associations with AD, like TREM2, cluster of differentiation 33 (CD33), and genes complement receptor 1 (CR1). TREM2 is a congenital immune recognition receptor with a conserved sequence expressed on the surface of microglia in the central system. It is involved in the regulation of microglial activation, proliferation and phagocytosis (Qin et al., 2021). TREM2 is believed to play a key role in the pathogenesis of AD. Several reviews have systematically reviewed the role of TREM2 in microglia function in AD (Ulrich et al., 2017; Qin et al., 2021). In addition, APOE can act as a ligand for TREM2 and the TREM2-APOE pathway acts as a regulator that can help restore homeostatic microglia (Krasemann et al., 2017). Okuzono et al. (2021) found that activation of TREM2 in AD may lead to anti-apoptotic signaling, immune responses and cytoskeletal alterations in microglia. On the contrary, CD33 inhibits anti-inflammatory signal transduction, cell adhesion and endocytosis of microglia, increasing the risk of AD (Ryan et al., 2017). Complement CR1 protein is a transmembrane glycoprotein expressed in microglia. The effect of CR1 on AD is mainly achieved by participating in the complement system to regulate the function of microglia (Zhu et al., 2022). Aβ and tau proteins bind to complement C3b and complement 1q (C1q), respectively, and then bind complement CR1 to activate microglia cells to respond to injury-related signals and exert clearance and protective effects (Medina and Avila, 2014). However, with the aggravation of AD, the over-activated CR1 will prompt microglia to produce superoxide, secrete inflammatory factors, and then damage neurons (Crehan et al., 2013).

### Microglia-Associated Inflammasome and Alzheimer’s Disease

Inflammasome, multiprotein complexes assembled with the participation of intracytoplasmic PRRs, is an important component of the natural immune system. Upon recognition of PAMPs or host-derived DAMPs, signals are transmitted to the immune system to initiate inflammation. Inflammasome promotes the maturation and secretion of pro-IL1β and pro-IL18 to produce IL-1β and IL-18 by causing the shearing and activation of Caspase-1 during the natural immune defense (Tastan et al., 2022). Several inflammasosomes have been identified, including NLRP1, NLRP3, NLRC4, and AIM2 (Cox et al., 2015; Friker et al., 2020). NLRP3 protein is the most characteristic inflammasome in neurodegeneration. Studies have shown that tau hyperphosphorylation and Aβ aggregation both lead to activation of NLRP3 inflammasome in microglia (Stancu et al., 2019; Friker et al., 2020). However, inhibition of the NLRP3 inflammasome in AD mouse models reduces microgliosis, suggesting that activation of the NLRP3 inflammasome in microglia may be the driver of its neurotoxic effect factors (Lonnemann et al., 2020; Shippy et al., 2020). Similarly, after the use of the NLRP3 inflammasome inhibitor MCC950, the clearance of Aβ in APP/PS1 transgenic mice was significantly increased, and the tau pathology was significantly reduced, indicating again that the NLRP3 inflammasome plays an important role in AD (Swanson et al., 2019). The AIM2 inflammasome, a member of the pyhin family, is also expressed in astrocytes and microglia, and is further up-regulated in glial cells in a mouse model of chronic neurodegeneration (Cox et al., 2015).

### Clearance or Aggravation of Aβ

In sporadic AD, inefficient Aβ clearance is the main pathogenic pathway. The Aβ pathological response activates microglia. In the early stages of AD, the phagocytosis of microglia contributes to the clearance of Aβ plaques, thereby slowing the progression of AD (Richter et al., 2020; Zhou et al., 2020). Specifically, Aβ plaque formation rapidly recruits microglia to participate in phagocytosis. TREM2 is essential for microglia to detect and respond to neurodegenerative signals and is regarded as an Aβ ligand, transducing Aβ-associated physiological and AD-related pathological effects (Zhao et al., 2018). TREM interacts directly with several forms of Aβ and has the strongest affinity for soluble Aβ_42_ oligomers (Lessard et al., 2018). The expression rate of TREM2 correlates with the rate of phagocytosis (Qin et al., 2021). *In vitro*, loss of TREM2 can result in decreased phagocytosis of apoptotic neurons, cellular debris (Kleinberger et al., 2014; Gawish et al., 2015). *In vivo*, microglia isolated from TREM2-deficient mice exhibits insufficient phagocytosis (Keren-Shaul et al., 2017). And human stem cell-derived microglia-like cells phagocytize fewer Aβ plaques than wild-type cells after loss of TREM2 (Claes et al., 2019). In addition, microglial activation is dependent on certain chemokines as well as receptors. TLR4 regulates downstream molecules NF-κB signaling and transcription activator 6 (Stat6) to induce microglia to shift to a favorable phenotype and improve their phagocytosis and clearance. In addition, TLR4 can promote phagocytosis and clearance of Aβ by upregulating the expression of CD14, scavenger receptor A (Su et al., 2016; Qin et al., 2018). The C-C motif chemokine receptor 2 (CCR2) expressed in microglia is a protein that binds chemokines such as CCL2, CCL7, and CCL12. In APP/PS1 mice, increased expression of CCL2 and CCR2 produced amyloidosis. The deficiency of CCL2 and CCR2 is followed by reduced Aβ phagocytosis and amyloid clearance (Jorda et al., 2021). In the early stage of AD pathology, microglia also release insulin-degrading enzyme (IDE), proteins of the matrix metalloproteinase 9 (MMP9), plasminogen and neprilysin, which are involved in Aβ degradation and induce Aβ clearance (Gao et al., 2018; Corraliza-Gómez et al., 2022). Another study with AD mouse models through high-resolution confocal and *in vivo* two-photon imaging found that microglia appear to have another neuroprotective mechanism, namely Aβ plaques are tightly wrapped by microglial processes, constituting a barrier that prevents the outward expansion of the plaque and the formation of neurotoxic fibril Aβ_42_ around the plaques (Condello et al., 2015).

However, with the progression of AD, the phagocytosis and degradation capacity of microglia gradually became insufficient compared with the continuous accumulation of Aβ, resulting in an increase in the accumulation of Aβ (Condello et al., 2015). The large amount of Aβ promotes the polarization of microglia toward the M1 phenotype (Xie et al., 2020). Aβ interacts with receptor complexes expressed on microglia, such as CD36 and TLR4, leading to the production of pro-inflammatory cytokines, chemokines, and neurotoxins. In turn, these cytokines down-regulate Aβ phagocytic receptors and Aβ degrading enzymes (Su et al., 2016), further promoting the development of AD. Yet inhibiting the production of inflammatory cytokines such as IL-1β resets the phagocytosis of microglia. The study by Heneka et al. (2013) showed that NLRP3/caspase-1 inflammasome activation reduced the phagocytic ability of microglia for Aβ.

### The Proliferation of Tau Protein

Soluble tau has been shown to be abundant both intracellular and in brain interstitial fluid. Misfolded aggregated forms of extracellular tau can also be taken up by neurons through endocytosis and readily induce misfolding and aggregation of intracellular tau (Yamada et al., 2014; Luo et al., 2015). Microglia have been shown to co-locate with Aβ plaques and NFTs, although the exact role involved is not fully understood (Serrano-Pozo et al., 2011, 2013). Microglia were found to be associated with tau proliferation, and the activation of microglia precedes the formation of NFTs (Maphis et al., 2015). Based on neuroimmune theory, activation of microglia releases pro-inflammatory mediators and ultimately promotes hyperphosphorylation of Tau protein and aggregation into NFTs (Maccioni et al., 2018). Evidence shows that tau protein released by neurodegenerative neurons can reactivate microglia, creating a vicious cycle in the pathway of neuronal degeneration (Zheng et al., 2002; Maccioni et al., 2018). In the study by Asai et al., depletion of microglia significantly inhibited tau proliferation. This involves exosomes secreted by microglia, which promote tau diffusion. Inhibition of exosome synthesis significantly reduces tau proliferation *in vitro/in vivo* (Asai et al., 2015). Luo et al. demonstrated that activated microglia can phagocytose and degrade p-Tau protein released from AD brains and effectively eliminate NFTs in P301S transgenic mice. And the use of small molecules or antibodies to stimulate phagocytosis and degradation of microglia may be a useful therapeutic strategy for tau immunotherapy (Luo et al., 2015).

Further studies showed that microglia-mediated tau phagocytosis may develop through interaction with the microglia receptor C–X3–C motif chemokine receptor 1 (CX3CR1), in which tau competes with the neuroligand CX3CL1. In contrast, CX3CR1-deficient microglia showed abnormal uptake and degradation of tau (Bolós et al., 2017). The microglia P2Y receptor (P2YR) is necessary for microglia to phagocytose neurons. In a recent study, microglia P2Y12 receptors were observed to be involved in the phagocytosis of full-length tau proteins via the lysosomal pathway (Chidambaram et al., 2022). Puigdellivol et al. found that knockout of the P2YR gene prevented intracerebral injection of Aβ in mice-induced microglial phagocytosis of neurons. *In vitro* experiments showed that the P2YR mediates Aβ and tau-induced neuronal and memory loss through microglial phagocytosis, suggesting that blocking the P2YR may contribute to the treatment of neurodegenerative diseases (Puigdellívol et al., 2021). In addition to being critical for the development and progression of Aβ pathology in mice, microglia and the NLRP3 inflammasome are equally important for tau pathology (Ising et al., 2019). Furthermore, alterations in APOE4 gene expression are often thought to be responsible for Aβ and tau-induced AD. Interestingly, the results of a mouse model of tau disease showed that APOE4 variants exacerbated tau pathology and involved upregulation of microglia responses and pro-inflammatory genes (Shi et al., 2017).

### Synaptic Pruning and Remodeling

Synaptic loss is associated with cognitive decline in AD. Microglia shape immature neuronal circuits by phagocytosis and elimination of inappropriate dendritic and axonal spines, known as synaptic pruning (Kulkarni et al., 2022). The impairment of microglia pruning mechanism leads to defects in synaptic development and neuronal connections. So how do microglia identify and molecularly target specific synapses for pruning? Studies have shown that they form postnatal neural circuits in a complement-dependent manner (Hong et al., 2016a; Shi et al., 2017). However, when microglia reach the damaged site, activated microglia promote phagocytosis through receptor recognition. Major scavenging receptors such as CD36 and macrophage receptor with collagen structure, (MARCO), low density lipoprotein (LDL) receptor, and three receptor tyrosine kinases are all involved in microglia phagocytosis (Kulkarni et al., 2022). Furthermore, the microglial receptors CR3 and C1q receptor (C1qR) are mainly involved in synaptic pruning during brain development, involving an increase in their binding to Aβ, leading to the degeneration of hippocampal synapses and loss of memory (Hong et al., 2016b). Conversely, CR3 deficiency enhances the protein degradation of Aβ, reduces neuronal loss, and increases cognitive function, especially in AD pathology (Czirr et al., 2017). Thus, unrestricted activation of complement signaling leads to synaptic degeneration and memory impairment. In addition, neurotrophic factors produced by microglia, especially microglia brain-derived neurotrophic factors, could stimulate and enhance synaptic activity (Mufson et al., 2019).

## Iron Metabolism in Alzheimer’s Disease

### Brain Iron

Iron, the most abundant transition metal in the brain, is an indispensable but potentially toxic trace element. As the highest oxygen-consuming organ in the human body, the brain has a high demand for iron. In the CNS, iron is found in neurons and glial cells. Iron is involved in a variety of very important processes in the brain, including the tricarboxylic acid cycle, myelination, myelin maintenance, and the synthesis of neurotransmitters. So, tight regulation of iron metabolism is essential to ensure neuronal homeostasis (Ashraf et al., 2019b).

Iron uptake, distribution, and sequestration are regulated at the cellular level by proteins such as transferrin, its receptor (TfR), and ferritin. Normally, dietary iron is present as heme iron and non-heme iron (Wang and Wang, 2019). The absorption of heme iron is not clear, while non-heme iron is transferred to the intestinal epithelial cell membrane or macrophages via divalent metal transporter 1 (DMT1) to transfer Fa^2+^ (Ingrassia et al., 2019). After its conversion to Fa^3+^ by hephaestin and/or copper cyanobacteria, it is exported out of the cell through the ferroportin 1 (FPN1) channel (Wang and Wang, 2019). However, the presence of the BBB prevents the direct entry of iron ions from the blood into the brain, which needs to be transferred through a special transporter system. Brain microvascular endothelial cells (BMVEC), astrocytes, microglia and pericytes together constitute the BBB (Rosenblum and Kosman, 2022). Then, how do iron ions pass indirectly through the BBB into the CNS? This involves some proteins in the BBB lumen and the abluminal or membrane closest to the interstitium. Iron ions enter the BMVEC from the lateral parietal surface of the blood and then cross the lateral surface of the basolateral membrane into the brain interstitium (Khan et al., 2018; Rosenblum and Kosman, 2022). In BMVECs, the non-transferrin-bound iron uptake (NTBI) pathway and the transferrin-bound iron (TBI) pathway are considered to be the main modes of iron transport into the brain (Khan et al., 2018; Rosenblum and Kosman, 2022). The majority of Fa^3+^ is taken up via TBI. Firstly, transferin transports Fa^3+^ to the surface of the luminal apical membrane of the cerebral microvascular system, where it binds to the TFR1 on the membrane and forms endocytic vesicles by endocytosis. Subsequently, Fa^3+^ dissociates from transferin and is reduced to Fa^2+^ (Khan et al., 2018). In the NTBI pathway, the reduced ferrous iron is carried into the cell by the divalent metal ion transport protein ZIP8 (gene SLC39A8) or ZIP14 (SLC39A14) (Rosenblum and Kosman, 2022). After iron enters the cytoplasm of these cells and meets metabolic needs, excess iron is stored in the cytoplasmic and mitochondrial ferritin (MTFT) or exported to the bloodstream via FPN1 (Ashraf et al., 2018; Wang and Wang, 2019).

### Iron Metabolism and Alzheimer’s Disease

The importance of iron to normal neural activity is well established, while disturbances in iron homeostasis contribute to the etiology of various neurodegenerative diseases, including AD. Evidence shows that iron accumulation exists in multiple regions of the AD brain (Gong et al., 2019), and increased iron concentration is found in both senile plaques and NFTs in AD (Zhu et al., 2009; Du et al., 2018). Brain iron accumulation has become a pathological feature of early AD. As for the mechanism of AD iron accumulation, increased BBB permeability and neuroinflammation are important reasons. Iron ions in the brain tend to accumulate with age. BBB permeability is altered in response to inflammatory stimuli, and iron transporter proteins on the membrane are upregulated, resulting in increased iron uptake and iron accumulation in the brain. The excessive accumulation of iron ions in turn further increases the BBB permeability, enhances inflammation, and affects the redistribution of iron ions in the brain, which in turn alters brain iron metabolism (Brian et al., 2018).

Iron accumulation accelerates the deposition of Aβ plaques (Ayton et al., 2020). It has been reported that the translation of the APP is regulated by cellular iron levels (Peng et al., 2021). α-secretase and β-secretase activation is particularly critical to attenuate or increase the formation of Aβ and its accumulation in the brain (Tian et al., 2022). The process is regulated by furin, whose transcription is regulated by intracellular iron concentration. Furin decreases in iron excess concentration, thereby increasing β-secretase activity and enhancing Aβ production (Choi et al., 2021; Peng et al., 2021). In addition, iron may regulate APP processing through IRPS/IRE (Peng et al., 2021). APOE4 has been found to inversely regulate the effects of iron on brain function and to regulate iron homeostasis proteins such as ferritin, increasing the risk of AD (Wood, 2015; Kagerer et al., 2020). Ferritin has been reported to be closely associated with APOE levels in cerebrospinal fluid and increases with the elevation of the gene APOE4. In APOE carriers, ferritin levels in the cerebrospinal fluid are elevated, suggesting altered iron metabolism and increased iron retention (Ayton et al., 2015). The translation of APP is affected by the IRE, and APP can indirectly regulate neuronal iron content and promote iron export by maintaining the stability of FPN (Rogers et al., 2002). Tau proteins are able to transport APP to the cell membrane and stabilize the FPN1-APP complex (Belaidi et al., 2018). Furthermore, iron promotes the formation of oligomeric tau, and overload iron promotes the hyperphosphorylation of tau protein (Soeda et al., 2015; Wan et al., 2019). However, a recent large cohort study has proposed the latest hypothesis of iron in AD: iron as an effector of neurodegeneration, has an additional downstream role, independent of tau or amyloid lesions, that is, iron-dependent ferroptosis is the relevant mechanism of neurodegeneration. Although such studies are gradually increasing, it is clear that a large amount of clinical trial data is needed to verify (Ayton et al., 2021). Taken together, iron can affect the progression of AD by regulating APP, Aβ, and hyperphosphorylated tau proteins.

## The Role of Microglia in Iron Metabolism in Alzheimer’s Disease

### Iron in Microglia

During aging and neurodegeneration, there is evidence of microglia proliferation, an increase in number, accompanied by phenotypic and functional changes (Srinivasan et al., 2016). During this process, microglia may become hyperreactive, which may be manifested specifically by dysregulated expression of cell surface receptors, increased release of pro-inflammatory cytokines, and loss of phagocytosis and the ability to degrade excess proteins. The resulting inflammation is thought to contribute to neurodegenerative diseases (Labzin et al., 2018). A prominent feature of neuroinflammation is increased extracellular iron activation and acquisition, followed by down-regulation of iron-interacting proteins, leading to intracellular iron isolation (Thomsen et al., 2015). Iron is closely related to immunity as it plays a key role in the proliferation and maturation of immune cells. To maintain iron homeostasis, innate immune cells, including microglia and astrocytes, sequester iron intracellularly by associating with cytosolic and mitochondrial ferritin. However, excess ROS and inflammation can be generated when this sequestration is disrupted (Abreu et al., 2018). Glial cells express various iron transporters and iron metabolism proteins, which play an important role in maintaining iron homeostasis and normal physiological functions of the brain (Healy et al., 2017; Song et al., 2018). In primary cultures of neurons, astrocytes, or microglia, microglia were found to be the most efficient in accumulating iron. When exposed to an iron environment, microglia retained three times more iron than neurons, suggesting that microglia have a greater capacity for iron accumulation and storage (Bishop et al., 2011). Interestingly, in another study, the highest concentration of iron was found in oligodendrocytes (5-fold higher iron concentration in oligodendrocytes compared to neurons) (Reinert et al., 2019). Unlike astrocytes, microglia exhibit ferritin immunopositivity because they contain mainly light chain ferritin, which can store iron, and are adept at scavenging iron (Ashraf et al., 2019a). Iron accumulation has been observed in microglia in the frontal cortex of patients with AD, which are usually located around Aβ plaques (van Duijn et al., 2017). Furthermore, iron-positive microglia were found in the hippocampus of AD patients but not in healthy control tissues (Zeineh et al., 2015). These results suggest that neuroglia are essential in maintaining iron homeostasis and normal physiological functions in the brain.

### Microglia Regulation of Iron Metabolism

**FIGURE 2:**
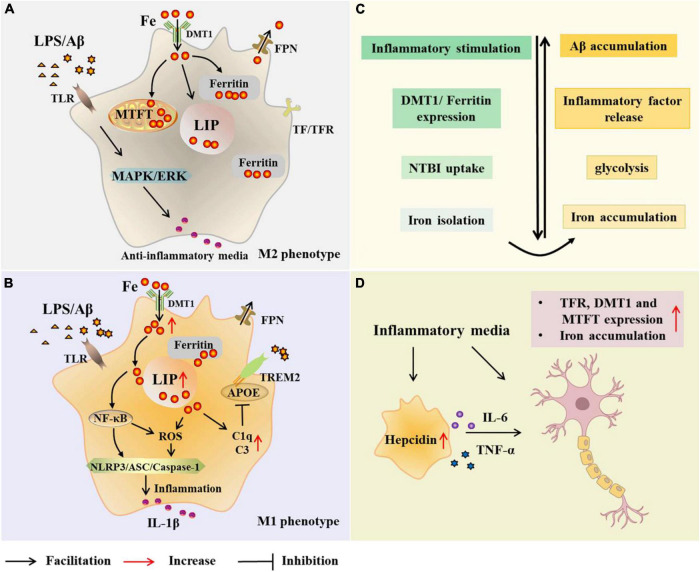
Microglial modulations on iron metabolism in Alzheimer’s disease (AD). In the case of AD, microglia are exposed to elevated iron levels, LPS, as well as the extracellular Aβ released from damaged neurons. The expression of DMT1 and ferritin is up-regulated in M2 microglia, which preferentially increases NTBI uptake, expands iron storage in ferritin and mitochondrial ferritin, and isolates extracellular and intracellular iron **(A,C)**. With high iron intake, microglia LIP increases, and iron and its induced ROS activate inflammasome, releasing inflammatory factors and promoting neuroinflammation **(B)**. Iron upregulates C3 and C1q expression and inhibits the APOE-TREM2 axis, leading to reduced phagocytosis of Aβ plaques **(B)**. Iron accumulation increases TNF-α expression and glycolysis, and reduces microglial phagocytosis **(C)**. Microglia alter neuronal iron homeostasis in neuroinflammation. Increased hepcidin in microglia and released inflammatory factors promote iron accumulation in neurons by affecting iron uptake and storage **(D)**. AD, Alzheimer’s disease; Aβ,amyloid beta-protein; LPS, lipopolysaccharide; TLR, toll-like receptor; DMT1, divalent metal transporter 1; FPN, ferropoiin; C1q, complement 1q; TfR, transferrin receptor; NTBI, non-transferrin-bound iron; MTFT, mitochondrial ferritin; IFN-γ, interferon γ; NF-κB, nuclearfactor-kappa B; ROS, reactive oxygen species; MAPK, mitogen activated protein kinase; ERK, extracellular regulated protein kinases; APOE, apolipoprotein E; TREM2 triggering receptor expressed on myeloid cells 2; ASC, apoptosis-associated speck-like protein containing a CARD; NLRP3, NLR family pyrin domain containing 3; TNF-α, tumor necrosis factor-α; IL-6, interleukin-6.

To understand the relationship between microglia and iron, it is first necessary to identify the major proteins and pathways of iron acquisition in microglia, and how iron uptake and metabolism are regulated when microglia are exposed to pro- or anti-inflammatory stimuli (Figure 2). Studies have shown that proteins involved in iron metabolism in microglia include TfR, DMT1, ferritin, and FPN (Urrutia et al., 2013; Thomsen et al., 2015). The NTBI uptake pathway and the canonical TBI uptake pathway are two pathways for cells to obtain iron. A correlation between microglia polarization, DMT1 expression and microglia iron uptake is known (Urrutia et al., 2013). Increased iron in microglia leads to phagocytosis and polarization, resulting in a harmful M1 phenotype (Kroner et al., 2014). Hepcidin is a hormone secreted by liver cells under inflammatory conditions and is known to modulate ferritin expression after translation. Interestingly, hepcidin can enter inflammatory brain regions with impaired BBB and affect ferritin function (Thomsen et al., 2015). In a recent study, Chaudhary et al. explored whether hepcidin in the brain contributes to iron accumulation in AD. The results showed that after the increase of hepcidin, the expression of FPN was decreased, the expression of ferritin and the total iron content increased (Chaudhary et al., 2021). In addition, IL-6 was elevated in Braak III-VI of AD, accompanied by microglial activation and amyloid plaque deposition. It may be that Aβ -induced activation of microglia increases the synthesis and secretion of IL-6 in Braak III-VI, leading to up-regulation of hepcidin (Chaudhary et al., 2021). However, this study is limited in that these observations were restricted to the cingulate cortex. Nonetheless, it is still relevant to facilitate further research. McCarthy et al. investigated transcriptional expression, protein levels, and iron transport in immortalized microglia treated with either pro-inflammatory stimulation (LPS or Aβ) or anti-inflammatory stimulation (IL-4). The results revealed that the transcription and protein expression of DMT1 and ferritin in immortalized microglia were up-regulated by inflammatory stimulation of LPS or Aβ, which preferentially increased NTBI uptake, expanded ferritin storage pools, and isolated extracellular and intracellular iron (McCarthy et al., 2018). Under these conditions, immortalized microglia increased glycolysis and extracellular acidification, supporting the changes of the microenvironment and facilitating the uptake of NTBI by DMT1. Response to pro-inflammatory mediators helps limit oxidative stress. Therefore, iron uptake under pro-inflammatory conditions is predominantly mediated by DMT1 (McCarthy et al., 2018). In addition, respiration was reduced in LPS-treated immortalized microglia, heme oxygenase-1 (HO-1) was induced, cell-free iron levels were increased, and labile iron pool (LIP) of microglia was increased. McCarthy et al. therefore suggested that microglia respond to inflammation by actively altering the iron status in their environment. Conversely, the anti-inflammatory cytokine IL-4 promotes the absorption of TBI due to increased TfR levels (McCarthy et al., 2018). And TfR is also involved in the regulation of microglia activation and enhances the phagocytosis of microglia (Carden et al., 2019).

Holland et al. found similar inflammatory, glycolysis, and iron-retaining phenotypes in microglia from APP/PS1 mice. Notably, increased intracellular iron concentration increased TNF-α expression and glycolysis in microglia, implying that increased intracellular iron concentration may drive metabolic and/or inflammatory changes (Holland et al., 2018). Similarly, in another experiment, McIntosh et al. (2019) induced inflammatory microglial phenotype with IFN-γ and Aβ in an APP/PS1 mouse model. Finally, IFN-γ stimulation increased DMT1 expression in microglia. Moreover, ferritin mRNA expression may be up-regulated and transferrin mRNA expression may be down-regulated, thereby promoting iron accumulation in activated microglia. Moreover, IFN-γ and Aβ increased glycolysis and glycolysis enzymes, including PFKFB3^+^ in microglia. Iron accumulation and glycolysis in microglia impaired its phagocytosis and weakened the clearance of Aβ (McIntosh et al., 2019). In addition, they previously found that FeCl_3_ could increase glycolysis. The correlation between inflammation, iron accumulation, and glycolysis was confirmed in the above data. Therefore, a novel idea was proposed that iron accumulation in microglia acts as a switch that pushes microglia into an inflammatory and glycolytic phenotype, prompting microglia to utilize less metabolically efficient glycolysis and limiting the ability of microglia to phagocytize Aβ (McIntosh et al., 2019). The current data, however, are insufficient to determine that correcting this metabolic shift can impair disease progression. Therefore, follow-up studies are needed.

The regulation of iron is regulated by a variety of cytokines. It has been reported that the secretion of non-fibrous p-tau protein by neurons in early AD promotes the activation of microglia and the release of cytokines such as IL-6 (Sanchez-Mejias et al., 2016; Newcombe et al., 2018). Anti-inflammatory factors released by microglia have been shown to negatively regulate iron metabolism, such as IL-10 (Huang et al., 2017). In addition, IL-6 and TNF-α may also alter iron homeostasis by affecting iron uptake and storage. TNF-α increases the expression of TfR1 and DMT1 by regulating the activity of IRP1, thus affecting the iron input of cells (Wang et al., 2013). In another study, treatment of hippocampal neurons, cortical astrocytes and microglia with IL-6, TNF-α and LPS increased the expression of DMT1 and decreased the expression of FPN1, resulting in increased iron uptake by hippocampal neurons and cortical microglia (Urrutia et al., 2013). On the other hand, there is new evidence that microglia are important regulators of neuronal iron homeostasis in neuroinflammation. A study reported that BV-2 microglia promoted iron accumulation in neurons and modulated the effects of inflammatory stimulation (LPS) on SH-SY5Y cells by changing iron uptake and storage conditions and cytokine secretion of SH-SY5Y cells (Pandur et al., 2018). Fractalkine is a potent inflammatory mediator expressed by neurons in the CNS and modulates microglia function by binding to the fractalkine receptor (CX3CR1). Fractalkine was found to increase hepcidin transcription and secretion in BV-2 microglia cells. Subsequently, increased hepcidin in microglia contributed to the iron accumulation of SH-SY5Y cells reduced cell viability via activating ferroportin internalization and DMT1, ferritin heavy chain and MTFT (Pandur et al., 2019).

### Effect of Iron Disturbance on Microglia

Microglia are influenced by iron levels while regulating iron metabolism. Aβ plaques in humans have been shown to be morphologically different from transgenic mouse Aβ plaques and to contain higher levels of iron and microglia (Meadowcroft et al., 2015a,b). Thus, increased iron content in the brains of AD patients may initiate and promote the formation of iron-rich Aβ plaques, leading to further microglial hyperplasia and toxicity. How do brain iron levels affect Aβ plaque formation, iron concentration in plaque, and microglial proliferation? A study of transgenic mouse brains subjected to iron overload and iron deprivation tests showed that increased brain iron levels accelerated plaque formation, promoted amyloid plaque senile morphogenesis, and activated microglia. In turn, increased brain iron levels were associated with the increase of plaque iron load and iron in microglia (Peters et al., 2018).

Excitotoxicity-induced neurodegenerative damage can lead to inflammation and the accumulation of iron-containing microglia. The microglia might act to scavenge excess iron originating from degrading neurons, glia and immunoreactive macrophages, but once beyond their capacity, iron accumulates in the neurons (Thomsen et al., 2015). Iron has been shown to induce mitochondrial damage, a danger signal that activates the inflammatory cascade by initiating the inflammasome (Nakamura et al., 2016). Iron itself and iron-induced ROS can activate NLRP3 inflammasome, causing neuroinflammation (Nakamura et al., 2016). In addition, iron activates NF-kB, which is also involved in neuroinflammation (Ashraf et al., 2018). LPS-activated microglia release cytokines, including TNF-α, IL-1α, and C1q, which are associated with autoimmune inflammation. The expression of APOE in microglia may be negatively affected by iron, resulting in reduced phagocytosis of the APOE-TREM2 axis and Aβ plaques, thereby disrupting tolerant immunity (Ulrich et al., 2018). On the other hand, iron up-regulates C3 and changes the expression of C1q, possibly preventing its binding with APOE (As shown in Figure 2; Li et al., 2015). Iron-ferritin dissociation has been found in animal models of AD, where LPS-induced ferritin phagocytosis increases intracellular free iron, leading to neuroinflammation (François et al., 2014; Biasiotto et al., 2016).

Although the effect of iron on microglia is well established, there is no definitive answer as to how iron affects microglia phenotype and function. Moreover, there are conflicting results regarding the activation phenotype of microglia after iron treatment. Several studies have shown that iron indirectly stimulates microglia to transform into a neurotoxic and pro-inflammatory M1 phenotype (Rao et al., 2006; Nnah et al., 2020). Neuromelanin can chelate with iron ions to form stable complexes (Raha et al., 2022). However, at excessive iron levels, neuromelanin-iron complexes are weakly bound in neurons, free iron ions damage neurons, and neuromelanin flows out of neurons cells. It is worth noting that neuromelanin and Aβ share similar characteristics in that they can both stimulate microglia to convert to the M1 phenotype and release neurotoxic factors like TNF-α and IL-6 (Rao et al., 2006). Additionally, in the experiment conducted by Nnah et al., immortalized microglial cells were incubated with Aβ with or without iron. The results showed that Aβ promoted the synthesis and secretion of IL-1β in immortalized microglia by activating the NF-κB signaling pathway. Iron appears to amplify the pro-inflammatory effect of Aβ, as microglia incubated with iron result in a higher pro-inflammatory response. Inhibition of DMT1 protects microglia against Aβ-induced inflammation. And ROS inhibitors antagonize Aβ-induced enhancement of IL-1β (Nnah et al., 2020). And iron deprivation experiments showed that iron deficiency not only prevented the induction of IL-1β and TNF-α by LPS, but also increased the expression of anti-inflammatory cytokine IL-10 and affected microglia function by promoting the release of inflammatory molecules (Rosato-Siri et al., 2018).

Other studies have found that iron inhibited the polarization and pro-inflammatory response of M1 macrophages induced by LPS or IFN-γ and conversely induced an anti-inflammatory M2 phenotype (Gan et al., 2017; Agoro et al., 2018). Notably, the data presented above came from mouse microglia and even mouse macrophages, whereas there are significant differences between human and mouse microglia and macrophages. The study by Kenkhuis et al. (2022) was the first to use human induced pluripotent stem cell-derived microglia (iPSC-MG) to examine the effects of increased iron levels on microglia under normal and inflammatory conditions. The results showed that iron treatment significantly affected the homeostatic function of iPSC-MG. However, it was nuclear factor E2-related factor 2 (Nrf2) and other oxidative stress pathways in microglia that were activated, rather than inducing the typical pro-inflammatory M1 or anti-inflammatory M2 activation (Kenkhuis et al., 2022). In another report by Kenkhuis et al., they investigated the occurrence of iron-accumulating microglia in the brains of patients with AD and its effect on their activation status. They identified a microglia subpopulation that exhibited increased expression of the iron storage protein ferritin light chain, decreased expression of the homeostatic markers TMEM119 and P2RY12 and a dystrophic morphology. And these cells were mainly found around Aβ plaques (Kenkhuis et al., 2021). These findings are consistent with a study by Yauger et al. (2019). They evaluated the effect of iron on LPS-activated microglia and its role in neuronal co-culture. The results showed a dose-dependent increase in ROS production by iron in LPS-treated microglia. However, no alteration of concomitant pro-inflammatory/M1 polarization markers was detected in microglia. Despite the absence of an altered microglial cell polarization phenotype, profound neurotoxic effects were present. Furthermore, this study demonstrated that NOX2 and NOX4 could be potential therapeutic targets for the treatment of iron-induced microglia-associated inflammation and neurotoxicity (Yauger et al., 2019). The inconsistency between species needs to be emphasized here. This is because in this study they used immortalized rats rather than mouse microglia (Yauger et al., 2019).

## Microglia and Ferroptosis

Currently, the role of iron in AD and the potential role of pro-inflammatory programs in iron accumulation in the brain have been demonstrated. Ferroptosis described by Stockwell is an iron-dependent form of cell death, which is induced by the lethal accumulation of lipid-based ROS generated by free iron (Dixon et al., 2012; Hirschhorn and Stockwell, 2019; Reichert et al., 2020). Ferroptosis is immunogenic, and the affected cells release DAMPs that promote a series of inflammation-related responses and even cell death. Ferroptosis is often accompanied with inflammatory manifestations in neural tissue: proliferation aggregation of astrocytes and microglia. Immune inflammation is closely related to the regulation of ferroptosis molecules (Proneth and Conrad, 2019; Sun et al., 2020). Although research on the complex relationship between ferroptosis and inflammation is still in the preliminary stage, several systematic reviews of the known association between ferroptosis and immune inflammation have been undertaken (Proneth and Conrad, 2019; Sun et al., 2020). Therefore, here we only briefly review and outline the status of recent studies of microglia and ferroptosis in AD.

### Crosslinking of Ferroptosis and Immune Inflammation

Iron accumulation, one of the prominent features of ferroptosis, is thought to be a factor in exacerbating inflammation. Nrf2 as well as downstream signaling were found to be sensitive to ferroptosis and counteract ferroptosis by coordinating iron/metal metabolism, glutathione synthesis/metabolism (Kerins and Ooi, 2018). Through redox regulation, Nrf2 attenuates inflammation and local ROS accumulation. Moreover, Nrf2 represses NF-κB transcription and pro-inflammatory genes encoding IL-6 and IL-1β (Kobayashi et al., 2016). In addition to inflammation caused by infection, iron accumulation is associated with an increase in senescent cells within tissues. And iron accumulation makes aging tissues vulnerable to oxidative stress, which can lead to cellular dysfunction and ferroptosis. ROS accumulation is a major cause of ferroptosis. Excessive ROS can lead to neuronal damage and subsequent inflammation through oxidative stress injury (Sun et al., 2021). Studies have shown that iron inhibitors can reduce the levels of inflammatory cytokines and ROS *in vivo* (Zhang et al., 2019). A recent study suggested that ferrostatin-1 may alleviate angiotensin II (Ang II)-induced astrocyte inflammation and ferroptosis by inhibiting ROS levels and activating Nrf2/Keap1/HO-1 signaling pathway (Li et al., 2021).

Inflammation, immunity, and cell death are associated with impaired lipid metabolism. Lipid peroxidation plays a significant role in inducing ferroptosis. With the increase of lipid peroxidation, steatosis may develop into an inflammatory state. Several regulatory factors have been found to act as bridges between lipid peroxidation and inflammation. Wen et al. (2019) confirmed that ferroptotic cells release a DAMP, namely high mobility group box protein 1 (HMGB1), in an autophagy-dependent manner. Autophagy dependent histone deacetylase (HDAC) inhibits the acetylation activity of HMGB1. This results in the release of HMGB1 during ferroptosis, which acquires immune-stimulating properties and then activates inflammatory responses by binding to pattern recognition receptors, thereby promoting the activation of the innate and adaptive immune systems (Wen et al., 2019). In addition, AGER is a polyligand receptor that is required for HMGB1 mediated macrophage inflammatory response to ferroptotic cells. Inflammatory responses to ferroptosis might be mediated by activation of the HMGB1-AGER signaling pathway (Wen et al., 2019). In addition to being a guardian of antioxidant damage, GPX4 is also an important participant in membrane repair, inflammation and ferroptosis suppression. GPX4 reduces ROS levels via regulating NF-κB and arachidonic acid oxidation, and blocks the production of pro-inflammatory lipid mediators, thereby effectively mitigating inflammatory damage (Li et al., 2019). Conversely, downregulation of GPX4 induces ERK activation and aggravates inflammation (Chen et al., 2015).

### Microglia and Ferroptosis in Alzheimer’s Disease

As reported by Kristin et al., Aβ_25–35_ induced intense ROS production in BV2 microglia via NADPH oxidase. Intracellular iron consumption inhibited ROS induced by Aβ_25–35_ (Part et al., 2015). In a study of the 5xFAD mouse model of AD, HO-1 was predominantly located to be expressed in microglia rather than other brain cells. With age, HO-1 expression in microglia increased 2_–_3 times in aged 5xFAD mice. Microglia overexpressing HO-1 were mainly found around Aβ plaques (Fernández-Mendívil et al., 2020). Fernández-Mendívil’s findings demonstrate that overexpression of HO-1 in microglia under inflammatory conditions can lead to toxic accumulation of iron, increase ROS, decrease GPX4 expression, and lead to ferroptosis and memory impairment. Conversely, HO-1 gene deletion in microglia, inhibition of HO-1 activity, or use of iron iron-chelating agents may have beneficial effects. This suggests that strategies that inhibit HO-1 activity or apply moderate iron chelating agents may be potential treatments to delay the progression of neurodegenerative disease (Fernández-Mendívil et al., 2021). One study suggested that iNOS expression may be a regulatory pathway for the pro-inflammatory resistance of M1 macrophages and microglia to the ferroptosis program (Kapralov et al., 2020). They assessed the sensitivity of macrophages and microglia to RSL3-induced ferroptosis by genetically regulating iNOS expression. It was found that knockdown of iNOS increased the susceptibility of macrophages and microglia to RSL3-induced death, whereas ferrostatin-1, a ferroptosis inhibitor, effectively inhibited this phenomenon. Similarly, iNOS inhibitors enhanced the sensitivity of activated macrophages, microglia, and primary microglia to RSL3-induced ferroptosis, but in the M1 rather than the M2 state. Therefore, iNOS might be an effective target for regulating ferroptosis and inflammation (Kapralov et al., 2020).

## Conclusion and Prospects

The relationship between microglia, iron and immune inflammation is undoubtedly close, and all three are either beneficial or detrimental to the disease. Data from studies on microglia in disorders of iron metabolism have certainly expanded their value in the pathogenesis and therapeutic strategies of AD. Iron is a double-edged sword and the body needs to precisely regulate intracellular iron levels and achieve homeostasis between iron uptake, recycling and storage. The role of microglia in neuronal iron metabolism is heavily dependent on the factors they release including neurophilic factors, pro-inflammatory factors, and lactoferrin. On the other side, elevated iron levels in microglia-activated areas may affect their function. Therefore, a deeper understanding of the interactions between microglia, iron and immune inflammation is necessary, which may help to explore therapeutic strategies by restoring brain iron homeostasis. For example, lutein increased the secretion of the anti-inflammatory cytokine IL-10 and increased the expression of MTFT in BV-2 cells (Pap et al., 2021). Aspirin significantly down-regulated the expression of TfR1 and up-regulated the expression of FPN1 and ferritin in BV-2 microglia *in vitro*. It indicated that aspirin negatively affects cellular iron content and partially reverses LPS-induced disruption of cellular iron homeostasis under inflammatory conditions *in vitro* (Xu et al., 2015).

Although microglia have several important functions, they do not operate in isolation. Recently, novel roles in the interaction of astrocytes and microglia in brain homeostasis have been demonstrated. For example, astrocyte-microglia interactions via complement activation play an important role in amyloid pathology in primary glial cells, hippocampal and cortical regions of APP transgenic mice (Lian et al., 2016). Other studies have shown that M1/M2 microglia polarization regulates the communication between astrocytes and microglia. And the induction of Aβ affects microglia-astrocyte interaction by altering microglia polarization (Xie et al., 2020). However, much of the current understanding of microglia interactions with other cells is discussed through cell-specific studies involving a great deal of speculation (Damisah et al., 2020). Therefore, more experimental proof is needed. Additionally, the critical performance of microglia at the transition from protective to pathogenic and how microglia recognize the effects of these changes on cell function remain to be solved. We can speculate from the available studies that microglia may play a role in ferroptosis. However, it is intriguing to know exactly what role microglia play in the process of ferroptosis. Finally, we need to emphasize that there are considerable differences between animal models and humans with AD in iron concentration, immune-inflammatory response, and structural morphology associated with Aβ plaques. Therefore, when transferring from animal experiments to human trials and when analyzing the data, the differences between the two must be noted and considered.

## Author Contributions

H-ZL completed the conception and wrote the first draft of the manuscript. Z-WZ, YC, H-YL, F-JL, and S-GX contributed to literature retrieval. All authors contributed to the manuscript revision, read, and finally the submitted version was approved by L-CG.

## Conflict of Interest

The authors declare that the research was conducted in the absence of any commercial or financial relationships that could be construed as a potential conflict of interest.

## Publisher’s Note

All claims expressed in this article are solely those of the authors and do not necessarily represent those of their affiliated organizations, or those of the publisher, the editors and the reviewers. Any product that may be evaluated in this article, or claim that may be made by its manufacturer, is not guaranteed or endorsed by the publisher.

## Abbreviations

AD: Alzheimer’s disease
A β: amyloid beta-protein
CR1: genes complement receptor 1
CD33: cluster of differentiation 33
TREM2: triggering receptor expressed on myeloid cells 2
APOE: apolipoprotein E
IRPs: iron regulatory proteins
NO: nitricoxide
BBB: blood-brain barrier
GWAS: genome-wide association studies
CNS: central nervous system
DAMPs: damage associated molecular patterns
PAMPs: pathogen-associated molecular patterns
TLRs: toll-like receptors
NOD: nucleotide binding oligomeric domain
NLRs: nucleotide binding oligomeric domain like receptors
DAM: disease-associated microglia
AIM2: absent in melanoma 2
IFN- γ: interferon γ
TNF- α: tumor necrosis factor- α
IL-6: interleukin-6
iNOS: inducible nitric oxide synthase
FGF: fibroblast growth factor
IGF-I: insulin like growth factor I
CCL2: chemokine (C–C motif) ligand 2
TGF- β: transforming growth factor- β
NF- κ B: nuclearfactor-kappa B
Stat6: NF- κ B signal transduction and transcriptional activator 6
NFTs: neurofibrillary tangles
APP: amyloid precursor protein
MIP-1 α: macrophage inflammatory protein 1
LPS: lipopolysaccharide
ASC: apoptosis-associated speck-like protein containing a CARD
NLRP3: NLR family pyrin domain containing 3
NOX: β-nicotinamide adenine dinucleotide phosphate oxidase
ROS: reactive oxygen species
TSPO: translocator protein
p38MAPK: P38 mitogen activated protein kinase
ERK: extracellular regulated protein kinases
CCR2: C–C motif chemokine receptor 2
IDE: insulin-degrading enzyme
MMP9: proteins of the matrix metalloproteinase 9
CX3CR1: C–X3–C motif chemokine receptor 1
Nrf2: subcellular localization of nuclear factor E2-related factor 2
MARCO: macrophagereceptor with collagen structure
LDL: low density lipoprotein
AGER: advanced glycosylation end-product-specific receptor
DMT1: divalent metal transporter 1
FPN1: ferropoiin 1
C1q: complement 1q
TfR1: transferrin receptor 1
MTFT: mitochondrial ferritin
IRE: iron-responsive mRNA element
NTBI: non-transferrin-bound iron
TBI: transferrin-bound iron
LIP: labile iron pool
HO-1: heme oxygenase-1
HDAC: dependent histone deacetylase
HMGB1: high mobility group box protein 1.
